# Bazi Bushen capsule improves the deterioration of the intestinal barrier function by inhibiting NLRP3 inflammasome-mediated pyroptosis through microbiota-gut-brain axis

**DOI:** 10.3389/fmicb.2023.1320202

**Published:** 2024-01-08

**Authors:** Shixiong Zhang, Mengnan Li, Liping Chang, Xinjing Mao, Yuning Jiang, Xiaogang Shen, Kunxu Niu, Xuan Lu, Runtao Zhang, Yahui Song, Kun Ma, Hongrong Li, Cong Wei, Yunlong Hou, Yiling Wu

**Affiliations:** ^1^College of Traditional Chinese Medicine, College of Integrated Traditional Chinese and Western Medicine, Nanjing University of Chinese Medicine, Nanjing, China; ^2^National Key Laboratory for Innovation and Transformation of Luobing Theory, Shijiazhuang, China; ^3^Key Laboratory of State Administration of TCM (Cardio-Cerebral Vessel Collateral Disease), Shijiazhuang, China; ^4^High-level TCM Key Disciplines of National Administration of Traditional Chinese Medicine—Luobing Theory, Shijiazhuang, China

**Keywords:** intestinal barrier function, microbiota-gut-brain axis, inflammasome, pyroptosis, SAMP8

## Abstract

**Purpose:**

The senescence-accelerated prone mouse 8 (SAMP8) is a widely used model for accelerating aging, especially in central aging. Mounting evidence indicates that the microbiota-gut-brain axis may be involved in the pathogenesis and progression of central aging-related diseases. This study aims to investigate whether Bazi Bushen capsule (BZBS) attenuates the deterioration of the intestinal function in the central aging animal model.

**Methods:**

In our study, the SAMP8 mice were randomly divided into the model group, the BZ-low group (0.5 g/kg/d BZBS), the BZ-high group (1 g/kg/d BZBS) and the RAPA group (2 mg/kg/d rapamycin). Age-matched SAMR1 mice were used as the control group. Next, cognitive function was detected through Nissl staining and two-photon microscopy. The gut microbiota composition of fecal samples was analyzed by 16S rRNA gene sequencing. The Ileum tissue morphology was observed by hematoxylin and eosin staining, and the intestinal barrier function was observed by immunofluorescence. The expression of senescence-associated secretory phenotype (SASP) factors, including P53, TNF-α, NF-κB, IL-4, IL-6, and IL-10 was measured by real-time quantitative PCR. Macrophage infiltration and the proliferation and differentiation of intestinal cells were assessed by immunohistochemistry. We also detected the inflammasome and pyroptosis levels in ileum tissue by western blotting.

**Results:**

BZBS improved the cognitive function and neuronal density of SAMP8 mice. BZBS also restored the intestinal villus structure and barrier function, which were damaged in SAMP8 mice. BZBS reduced the expression of SASP factors and the infiltration of macrophages in the ileum tissues, indicating a lower level of inflammation. BZBS enhanced the proliferation and differentiation of intestinal cells, which are essential for maintaining intestinal homeostasis. BZBS modulated the gut microbiota composition, by which BZBS inhibited the activation of inflammasomes and pyroptosis in the intestine.

**Conclusion:**

BZBS could restore the dysbiosis of the gut microbiota and prevent the deterioration of intestinal barrier function by inhibiting NLRP3 inflammasome-mediated pyroptosis. These results suggested that BZBS attenuated the cognitive aging of SAMP8 mice, at least partially, by targeting the microbiota-gut-brain axis.

## 1 Introduction

Aging is the cause of many age-related diseases, which can lead to gradual decline in body function and increase the risk of age-related diseases (Gavrilov and Gavrilova, 2017). One of the key components of the body that is affected by aging is the intestinal barrier, which consists of the intestinal epithelium that protects the body from the external environment and adapts to various stimuli, including aging (El Maï et al., 2023; Salazar et al., 2023). Many studies have shown that aging has a significant impact on the structure and function of the intestine (Branca et al., 2019). It has been suggested that impaired intestinal barrier function is a major sign of aging (Suzuki et al., 2022; El Maï et al., 2023). Furthermore, intestinal barrier dysfunction and the resulting pathological damage are believed to be important mechanisms underlying aging (Funk et al., 2020; Salazar et al., 2023). Therefore, maintaining the integrity of the intestinal barrier could be an effective way to delay aging.

The senescence-accelerated prone mouse 8 (SAMP8) is a widely used model for aging research (Roig-Soriano et al., 2022), especially for brain cognition (Cristòfol et al., 2012). Interestingly, some studies have found that the intestine of SAMP8 mice shows signs of aging before the brain does (Ben Othman et al., 2020; D'Antongiovanni et al., 2021). However, the exact changes that occur in the intestine of SAMP8 mice are still unclear. To explore this issue, we conducted a study on the intestinal aging process in SAMP8 mice. Previous studies have suggested that the aging of the gastrointestinal tract involves the degeneration of villi and the reduction of tight junction proteins, which are essential for maintaining the intestinal barrier (Soenen et al., 2016). However, the causes of intestinal barrier dysfunction due to aging are still unknown (Ma et al., 2020). Gut dysbiosis, or the imbalance of gut bacteria, can impair the intestinal barrier and trigger systemic inflammation, as shown by increased damage to the colon and liver and elevated levels of inflammatory markers (Haran and McCormick, 2021; Zhao et al., 2023). Moreover, endotoxins produced by gut bacteria, such as lipopolysaccharides (LPS), can also leak into the bloodstream (Li T. et al., 2023), which may activate the inflammasomes mediated by the NOD-like receptor family pyrin domain-containing 3 (NLRP3) pathway (Li Y. et al., 2023). Inflammasomes are protein complexes that regulate inflammation and cell death. Previous studies have shown that inhibiting the NLRP3 inflammasome can extend lifespan (Marín-Aguilar et al., 2020). Inflammasome activation can also induce pyroptosis, which is a type of cell death that involves inflammation and tissue damage (Zheng and Kanneganti, 2020; Barnett et al., 2023). Therefore, we hypothesize that NLRP3 inflammasome activation and pyroptosis may play roles in intestinal barrier damage caused by aging. We aim to test this hypothesis in SAMP8 mice.

Bazi Bushen (BZBS) is a traditional Chinese medicine (TCM) that contains 16 herbs (*Semen Cuscutae, Fructus Lycii, Epimedii Folium, Fructus Schisandrae Sphenantherae, Fructus Cnidii, Fructus Rosae Laevigatae, Semen Allii Tuberosi, Radix Morindae Officinalis, Herba Cistanches, Fructus Rubi, Radix Rehmanniae Recens, Radix Cyathulae, Radix Ginseng, Cervi Cornu Pantotrichum, Hippocampus,* and *Fuctus Toosendan*), including the seeds of eight plants and other herbs such as *ginseng* and *Cistanche*. BZBS contains a variety of bioactive compounds with potential anti-aging properties. These compounds include ginsenosides, polyphenols, and crude polysaccharides (Hao et al., 2022), which have been shown to regulate oxidative stress, apoptosis, inflammation, and the microbe-gut-brain axis. Additionally, *Cistanche deserticola* polysaccharides, one of the key anti-aging compounds in BZBS, have been shown to alleviate cognitive decline in aging model mice by restoring the gut microbiota-brain axis (Gao et al., 2021). Furthermore, Fructus Lycii, another important constituent of BZBS, has been demonstrated to alleviate age-related bone loss by targeting BMPRIA/BMPRII/Noggin (Sun et al., 2023). These findings suggest that BZBS has the potential to delay the aging process by targeting multiple pathways that contribute to age-related decline. It is based on the ancient theory of kidney-tonifying, which is believed to enhance vitality and longevity. BZBS has been shown to mitigate epigenetic aging and extend health span in naturally aging mice (Mao et al., 2023). Furthermore, BZBS has demonstrated beneficial effects on aging-related diseases in various organs, especially in improving cognitive function (Ji et al., 2022; Wang et al., 2023). However, the potential of BZBS to delay aging by repairing intestinal damage caused by accelerated aging has not been explored yet.

Here, we studied how BZBS affects the aging and damage of the intestines in SAMP8 mice, which are a model of accelerated aging. We discovered that SAMP8 mice had cognitive problems and severe intestinal damage as they got older, and they lost their normal intestinal barrier function. BZBS improved their overall health and cognition and restored their intestinal barrier function. BZBS also reduced inflammation and senescence, increased intestinal cell growth and differentiation, changed the gut microbiota composition, and prevented pyroptosis, a type of cell death caused by inflammasomes. Our results suggest that BZBS could slow down aging by reversing the aging and damage of the intestines.

## 2 Materials and methods

### 2.1 Animal models and drugs

The 8-week-old male SAMP8 mice were provided by Peking University Health Science Center, and senescence-accelerated mouse resistant 1 (SAMR1) mice were selected as the control group. The mice were kept at constant environmental conditions of 22 ± 2°C, 55 ± 10% relative humidity and a 12 h/12 h light/dark cycle. All mice were adaptively fed for 1 week. Then, the SAMP8 mice were randomly divided into four groups consisting of 10 mice each, including the model group, the BZ-low group, the BZ-high group and the RAPA group. The BZ-low group and the BZ-high group were given 0.5 g/kg/d and 1 g/kg/d BZBS, respectively. The RAPA group was given 2 mg/kg/d rapamycin. Medicated feed was adopted as a drug delivery method. BZBS was provided by Hebei Shijiazhuang Yiling Pharmaceutical Co., Ltd. The study lasted for 6 months. The animal protocol was approved by the Ethics Commission of the Hebei Yiling Chinese Medicine Research Institute (N2021087).

### 2.2 Hematoxylin and eosin staining

The mice were anesthetized by pentobarbital sodium and sacrificed. Then ileum tissue samples from each mouse were separated. And the samples were fixed with 4% paraformaldehyde. Then they were cut into slices with a thickness of 5 μm, and stained with hematoxylin and eosin (H&E). Finally, the pathological change was observed under a light microscope (Leica Microsystems, Wetzlar, Germany). The length of intestinal villi was calculated by Image Pro Plus 6.0 software. The protocol was performed as previously described (Xu et al., 2023).

### 2.3 Nissl staining

According to the manufacturer’s instructions, the Nissl staining kit (Beyotime, Shanghai, China) was used for staining (Ji et al., 2022). In brief, the sections were placed in toluidine blue at 60°C for 40 min. Then the sections were washed by distilled water. After being placed in 95% ethanol for rapid differentiation, the sections were transferred to anhydrous ethanol for rapid dehydration. Finally, the results were observed under a light microscope (×400 magnification).

### 2.4 Immunofluorescence staining

For immunofluorescence (IF), the ileum tissue samples were fixed in 4% paraformaldehyde, and then sectioned. The sections were repaired by microwave heating with 0.01 M sodium citrate antigen retrieval buffer. After incubated with hydrogen peroxide solution, the sections were sealed with 5% BSA at room temperature for 30 min. Then the sections were incubated with ZO-1 (Servicebio, GB111402, 1:500 dilution, China) and Occludin (Servicebio,GB111401, 1:500 dilution, China) overnight at 4°C. After being washed with PBS, the sections were incubated with Alexa Fluor 488 (Servicebio, GB25303, 1:500 dilution, China) and Coralite594 (Proteintech, SA00013-3-100, 1:300 dilution, China). Finally, images were captured with an inverted fluorescence microscope (Leica Microsystems, Wetzlar, Germany). Quantification of the positive area was performed using Image Pro Plus 6.0 software. The standard procedure was the same as that used in our previous study (Mao et al., 2023).

### 2.5 Immunohistochemistry staining

For immunohistochemistry (IHC), the sections were incubated with primary antibodies at 4°C overnight. Then, the reacted sections were incubated with the corresponding secondary antibody. After that, the sections were treated with the newly prepared DAB solution. Here, primary antibodies against F4/80 (Abcam, ab300421, 1:5000 dilution, United States), Ki67 (Abcam, ab15580, 1:1000 dilution, United States) and Lgr5 (Affinity, DF2816, 1:200 dilution, United States) were probed, respectively. Finally, the results were observed under a light microscope. All experiments followed the manufacturer’s instructions (Yang X. et al., 2020; Yang H. H. et al., 2020).

### 2.6 Real-time quantitative PCR

Total RNA from the ileum tissues was extracted using the TransZol Up Plus RNA Kit (TransGen Biotech, ER501-01, China) followed by cDNA synthesis using the GoScript™ Reverse Transcription System (Promega, A5001, United States). Then, the quantitative real-time PCR was performed using the MonAmp™ ChemoHS qPCR Mix kit (Monad, MQ00401S, China). All experiments followed the manufacturer’s instructions. The primers were shown in the Table 1. The standard procedure was the same as that used in our previous study (Ji et al., 2022).

**Table 1:** Sequences of primers used for qRT-PCR in this study.

Primer	Forward	Reverse
P53	CCCCTGTCATCTTTTGTCCCT	AGCTGGCAGAATAGCTTATTGAG
TNF-α	AGTGACAAGCCTGTAGCCC	GAGGTTGACTTTCTCCTGGTAT
NF-κB	GACACGAACAGAATCCTCAGCATCC	CCACCAGCAGCAGCAGACATG
IL-4	GGTCTCAACCCCCAGCTAGT	GCCGATGATCTCTCTCAAGTGAT
IL-6	TGTATGAACAACGATGATGCACTT	ACTCTGGCTTTGTCTTTCTTGTTATCT
IL-10	CCTCGTTTGTACCTCTCTCCG	AGGACACCATAGCAAAGGGC
IL-1β	CAGGATGAGGACATGAGCACC	CTCTGCAGACTCAAACTCCAC
IL-18	AGCTTGTGAAAAAGAGAGAGACCT	GCTAGTCTTCGTTTTGAACAGTGA
GAPDH	TGGATTTGGACGCATTGGTC	TTTGCACTGGTACGTGTTGAT

### 2.7 Western blotting assay

The total protein of ileum tissues for western blotting was extracted, and the protein concentration was determined by a BCA protein assay kit following the manufacturer’s instructions. Then the protein was separated by SDS-PAGE. After being separated, the protein was transferred onto PVDF membrane. Subsequently, the membrane was blocked with 5% BSA, and incubated with primary antibodies. Here, Cleaved caspase-1 (Affinity, AF4005, United States), NLRP3 (Servicebio, GB114320, China), Cleaved GSDMD (Abcam, ab255603, United States) and GAPDH (Servicebio, GB11002, China) antibodies were used and blots were quantified using ImageJ Software (NIH, Bethesda, MD, United States). Each group was tested using three samples. The results were normalized to the GAPDH band. The standard procedure was the same as that used in our previous study (Yang X. et al., 2020; Yang H. H. et al., 2020).

### 2.8 Enzyme-linked immunosorbent assay (ELISA)

According to the manufacturer’s instructions, the expression level of LPS in the liver was measured using the ELISA kit (Cusabio, CSB-E13066m, China). First, the standards and the samples were added to the reaction wells (100 μL/well), and then, a negative control was set up, incubated for 1.5 h. To each well, horseradish peroxidase (HRP)-labeled streptavidin (100 μL/well) was added, followed by 30 min of incubation. Then, the plate was incubated for 30 min in the dark with the chromogenic solution. After that, the termination solution was added to terminate the reaction. Eventually, the OD values of the wells were evaluated using a microplate reader (Beckman, Germany) at 450 nm.

### 2.9 *In vivo* two-photon imaging assay

To assay the permeability of the blood–brain barrier (BBB), the mice were anesthetized and placed on a head-fixing apparatus under a custom-modified two-photon microscope (Scientifica, Uckfield, United Kingdom). A dye mixture containing 12.5 mg/mL fluorescein isothiocyanate (FITC)-conjugated dextran (70 kDa molecular weight, Sigma-Aldrich, US) and 6.25 mg/mL tetramethylrhodamine (TRITC)-conjugated dextran (40 kDa molecular weight, Thermo Fisher Scientific, United States) was administered via tail vein injection. *In vivo* image stacks were then acquired. The protocol was performed as previously described (Lee et al., 2018).

### 2.10 16S rRNA gene sequencing

Fresh fecal samples of the three groups (the control group, the model group and the BZ-high group) were collected for 16S rRNA gene sequencing (*n* = 10). The procedure was performed as previously described (Bolyen et al., 2019).

### 2.11 Statistical analysis

The data were expressed as mean ± SD, and all statistical analyses and graphing were performed using SPSS 26.0 (SPSS, Armonk, United States) and GraphPad Prism 8.0.2 (GraphPad, San Diego, United States). Differences among groups were determined using the one-way analysis of variance (ANOVA), and Tukey’s test was used for *post hoc* comparisons. *p* < 0.05 was considered as a significant difference.

## 3 Results

### 3.1 BZBS improved the cognitive function in SAMP8 mice

**Figure 1:**
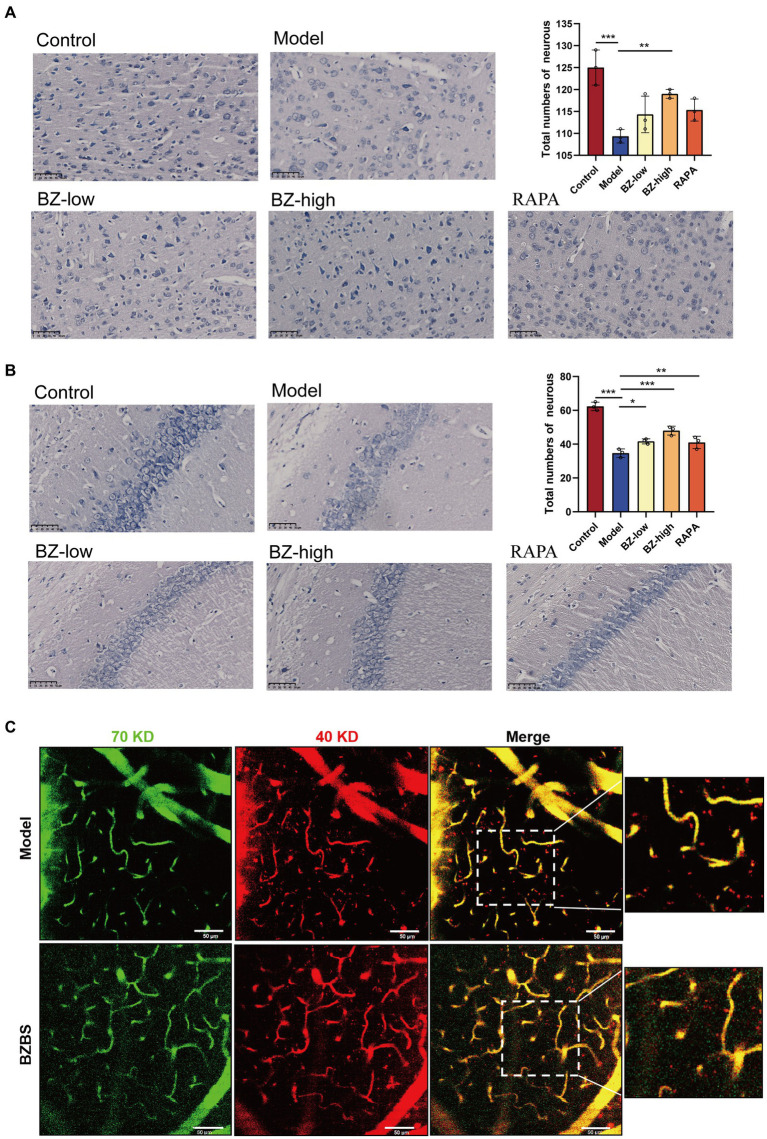
Effects of BZBS on the cognitive function in SAMP8 mice. **(A)** The result of Nissl staining in the cortex. **(B)** The result of Nissl staining in the hippocampus. **(C)** The result of *in vivo* two-photon imaging assay (^*^p < 0.05; ^**^p < 0.01; ^***^p < 0.001).

To measure the number of neurons, Nissl staining was used in our study. The results showed that the number of neurons in the cortex and hippocampus of the model group was significantly decreased compared with that in the control group. Furthermore, BZBS treatment increased the neuronal numbers in the SAMP8 mice (Figures 1A,B). To assay the integrity of the BBB in the SAMP8 mice, we performed *in vivo* two-photon imaging to detect extravasation in the cerebral cortex. We observed extravasation of 40 kDa TRITC-conjugated dextranin in the model group, signifying increased BBB leakage. Consistent with our hypothesis, BZBS treatment reduced the extravasation in the cerebral cortex (Figure 1C). These results fully reflected the protective effect of BZBS on the cognitive function in the SAMP8 mice.

### 3.2 BZBS ameliorated the intestinal barrier function in SAMP8 mice

**Figure 2:**
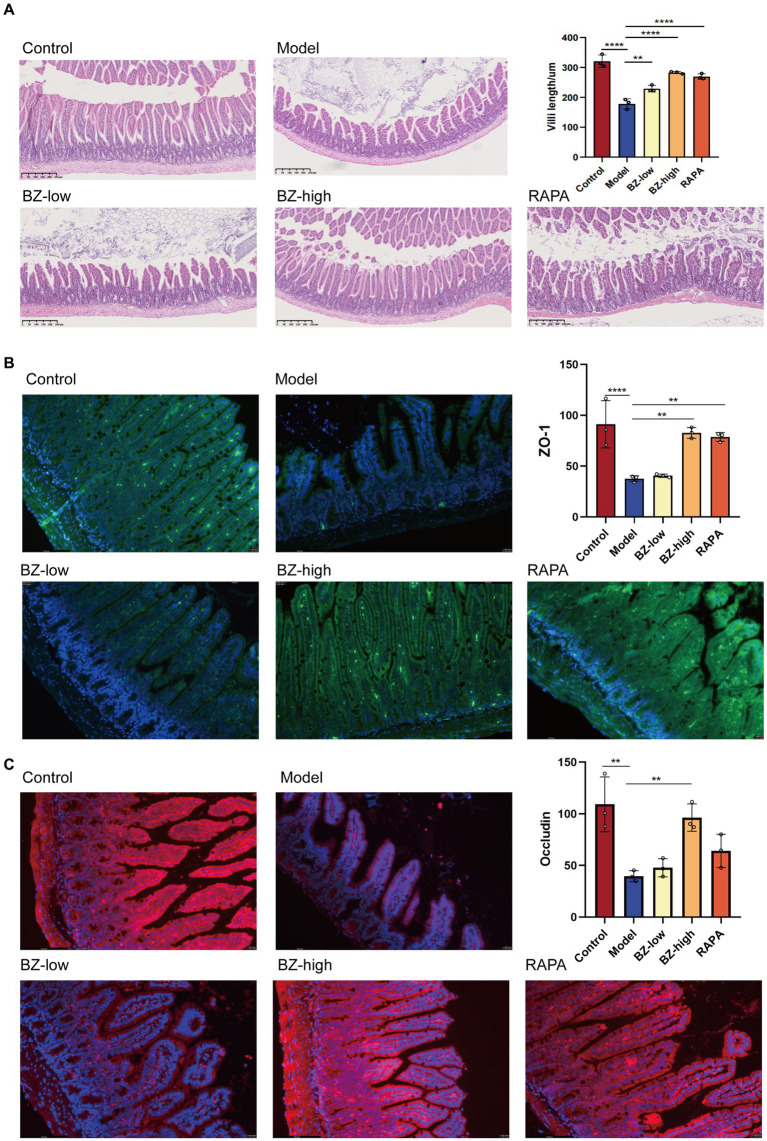
Effects of BZBS on the intestinal barrier function in SAMP8 mice. **(A)** The result of H&E staining. **(B)** The result of ZO-1 expression level by immunofluorescence staining. **(C)** The result of occludin expression level by immunofluorescence staining (^*^p < 0.05; ^**^p < 0.01; ^***^p < 0.001; ^****^p < 0.0001).

In our study, the morphological change was assessed by H&E and IF staining. The results of H&E staining showed that the intestinal barrier of SAMP8 mice was damaged with reduced amounts of intestinal villi in the model group when compared with this in the control group. And BZBS treatment could reduce damage to intestinal barrier function and repair intestinal villus structure (Figure 2A). Consequently, the increased protein expression of ZO-1 and occludin in ileum tissues of BZBS-treated mice were further confirmed by IF imaging, respectively, (Figures 2B,C).

### 3.3 BZBS inhibited the secretion of SASP factors and macrophage infiltration in SAMP8 mice

**Figure 3:**
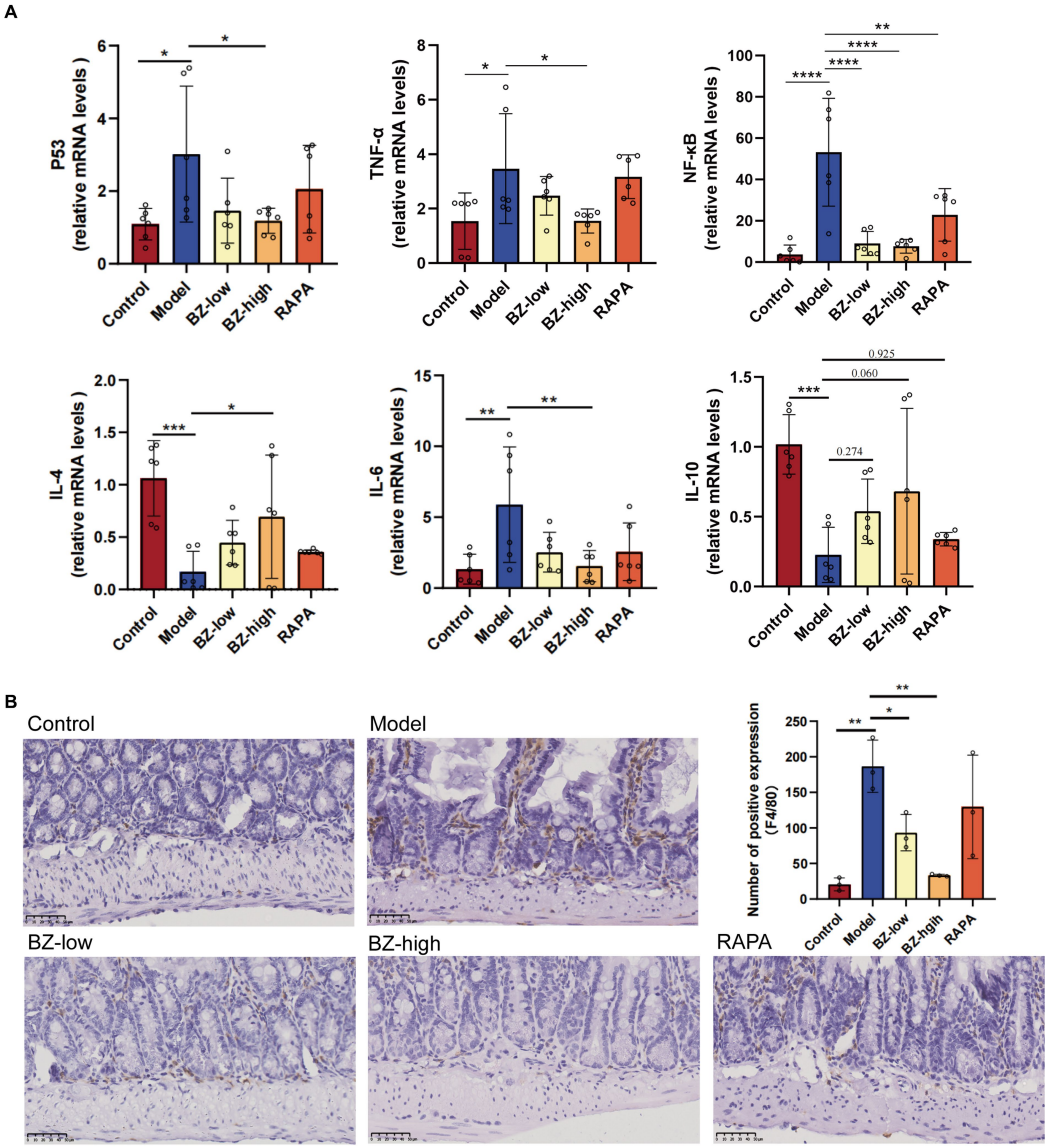
Effects of BZBS on the SASP in SAMP8 mice. **(A)** The result of mRNA expression level, including P53, TNF-α, NF-κB, IL-4, IL-6, IL-10. **(B)** The result of F4/80-positive macrophages in ileum sections in the SAMP8 mice (^*^p < 0.05; ^**^p < 0.01; ^***^p < 0.001; ^****^p < 0.0001).

In this study, the gene expression level of SASP markers, including P53, TNF-α, NF-κB and IL-6, all increased in the model group compared with the control group. In addition, the gene expression level of IL-4 and IL-10 decreased in the comparison between the model group and the control group. Moreover, these parameters were normalized by BZBS administration. These results clearly demonstrated that BZBS administration inhibited the secretion of SASP factors in the SAMP8 mice (Figure 3A). In addition, the sections were used to detect the expression level of F4/80 by IHC in the SAMP8 mice. The results indicated that F4/80 expression was increased in the model group. And these parameters were normalized by BZBS administration (Figure 3B).

### 3.4 BZBS contributed to the regulation of intestinal cell proliferation and differentiation in SAMP8 mice

**Figure 4:**
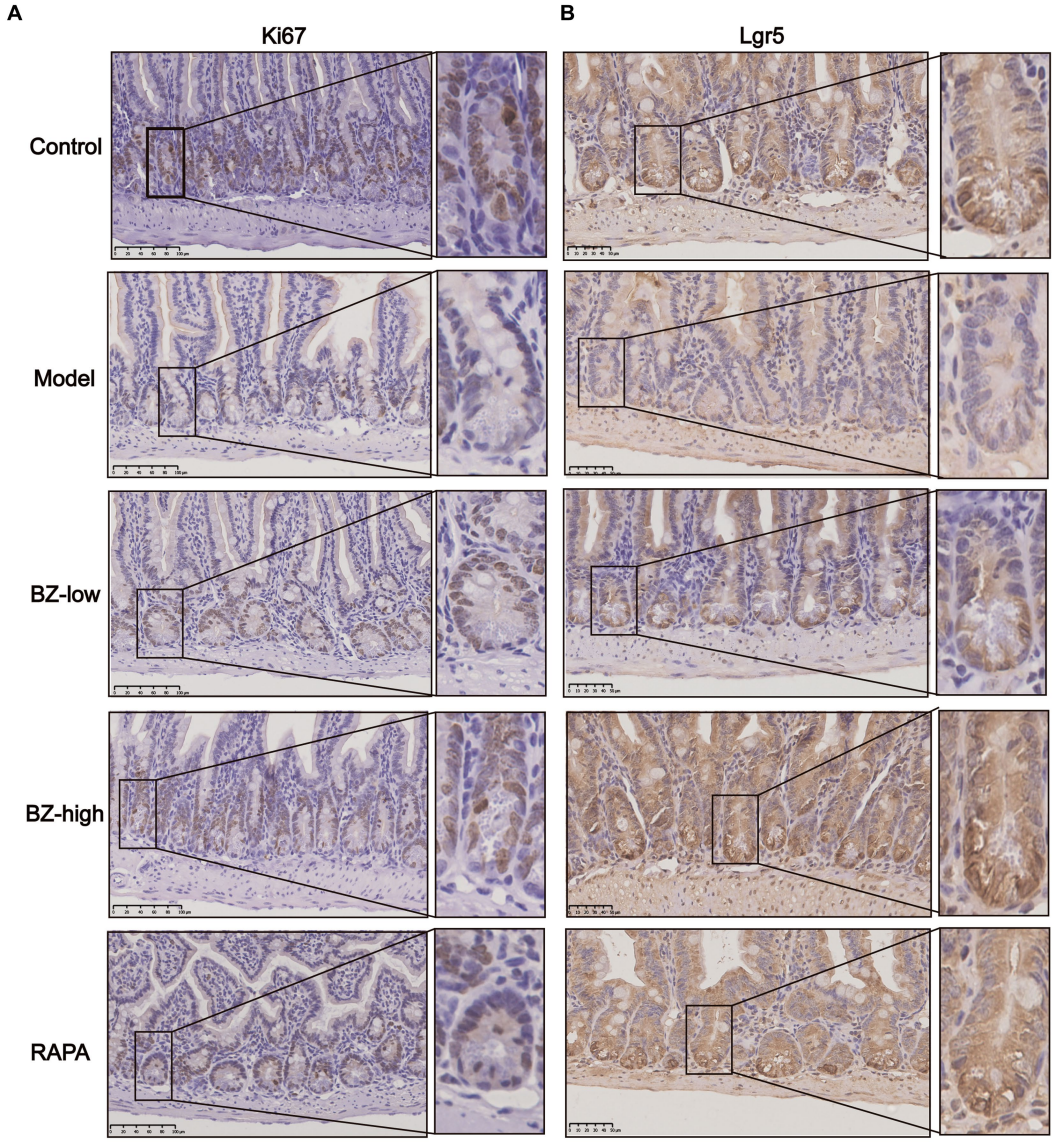
Effects of BZBS on regulation of proliferation and differentiation. **(A)** The result of proliferation level, and the molecular indicator of proliferation is Ki67. **(B)** The result of differentiation level, and the molecular indicator of proliferation is Lgr5.

Intestine undergoes a continual process of proliferation and differentiation. In our study, we observed decreased proliferation of intestinal cell in the model group as noted by decreased expression of Ki67. Moreover, these parameters were normalized by BZBS administration (Figure 4A). As BZBS promoted crypt cell proliferation, we postulated that BZBS might also play a role in regulating intestinal stem cell (ISC) activity. To investigate this hypothesis, we next determined the effect of BZBS on ISC marker. Compared with those in the control group, the levels of the ISC marker Lgr5 were downregulated in the model group. Moreover, these parameters were normalized by BZBS administration (Figure 4B). These results indicated that BZBS may contribute to the processes of intestinal cell proliferation and differentiation.

### 3.5 BZBS treatment altered gut microbiota composition in SAMP8 mice

**Figure 5:**
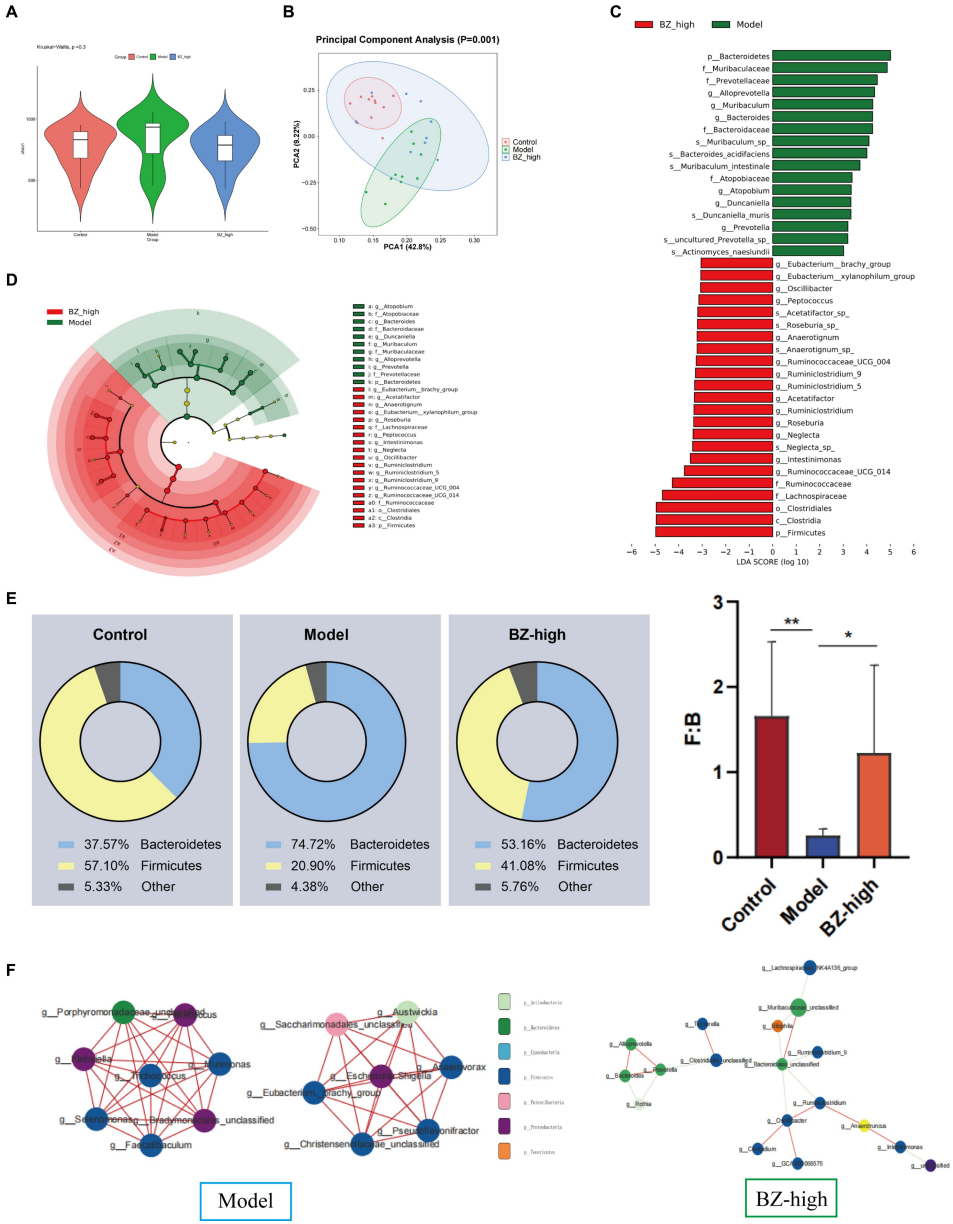
Effects of BZBS on theintestinal microbiota homeostasis in SAMP8 mice. **(A)** α diversity by Chao 1 index. **(B)** β diversity by PCA. **(C)** LDA score representing the taxonomic data with significant difference between the model group and the BZ-high group. **(D)** Taxonomic cladogram generated from LEfSe analysis of 16 s rRNA gene sequencing. **(E)** Results of comparison between Bacteroidetes and Fimicutes. **(F)** Results of dominant bacteria among different groups (^*^p < 0.05; ^**^p < 0.01).

In this study, we analyzed the effect of BZBS on the gut microbiome by 16S rRNA gene sequencing. There was no significant difference between the model group and the BZ-high group in α-diversity (Figure 5A), whereas β-diversity analysis showed significant differences in the composition and abundance of the microbiome (Figure 5B). Linear discriminant analysis effect size (LEfSe) analysis revealed that the phylum *Bacteroidetes*, the families *Atopobiaceae, Bacteroidaceae, Mutibaculaceae* and *Prevotellaceae*, the genera *Atopobium, Bacteroides, Duncaniella, Muribaculum, Alloprevotella* and *Prevotella* were enriched in the model group, whereas the phylum *Firmicutes*, the families *Ruminococcaceae* and *Lachnospiraceae*, the genera *Ruminnococcaceae_UCG_014*, *Ruminnococcaceae_UCG_004, Ruminiclostridium_9, Ruminiclostridium_5, Ruminiclostridium, Oscillibacter, Neglecta, Intestinimonas, Peptococcus, Roseburia, Eubacterium_xylanophilum_group, Anaerotignum, Acetatifactor* and *Eubacterium_ brachy_group* were enriched in the BZ-high group (Figures 5C,D). The relative abundance of *Firmicutes/Bacteroidetes* ratio was significantly decreased in the model group, while the relative abundance was increased in the BZ-high group (Figure 5E). In detail, *Firmicutes* was the dominant bacteria in the BZ-high group, while *Bacteroidetes* was the dominant bacteria in the model group (Figure 5F). These results indicated that the BZ-high group had a unique intestinal microbiome composition compared with the model group.

### 3.6 BZBS inhibited inflammasome-mediated pyroptosis to reduce the intestinal damage in SAMP8 mice

**Figure 6:**
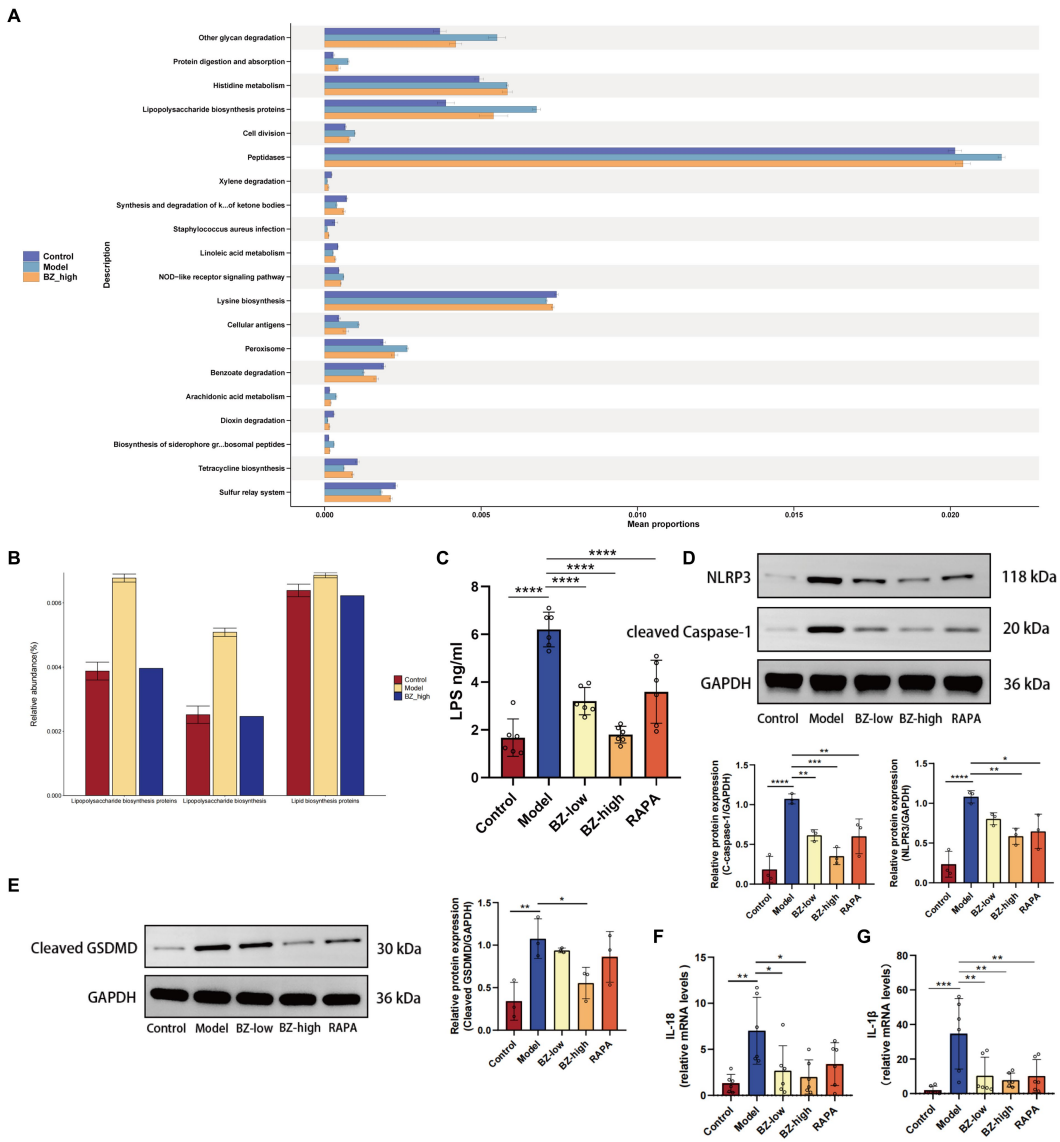
Effects of BZBS on inflammasome and pyroptosis in SAMP8 mice. **(A)** The result of KEGG enrichment analysis. **(B)** Comparison result of LPS related pathways. **(C)** The result of LPS level in the liver tissue. **(D)** The expression level of NLRP3 and cleared caspase-1 in the ileum tissue. **(E)** The expression level of cleaved GSDMD in the ileum tissue. **(F)** The result of mRNA expression level about IL-18. **(G)** The result of mRNA expression level about IL-1β (^*^p < 0.05; ^**^p < 0.01; ^***^p < 0.001; ^****^p < 0.0001).

To determine the mechanism of BZBS in prevention of intestinal barrier damage, the relative abundance of KEGG pathway was predicted by phylogenetic reconstruction of unobserved states 2 (PICRUSt2) and the functional change of gut microbiota after BZBS treatment was identified. The results revealed that lipopolysaccharide biosynthesis proteins were significantly enhanced in the model group (Figures 6A,B). To clarify the role of LPS in resulting in the intestinal damage, we tested the level of LPS in the liver by ELISA. The results indicated that LPS translocation due to intestinal barrier damage was reduced by BZBS (Figure 6C). Next, we measured the expression of NLRP3 and cleaved caspase-1, two markers of inflammasome activation, and the level of cleaved GSDMD, a marker of pyroptosis, in each group. As shown in the results, BZBS inhibited the expression of NLRP3 and cleaved caspase-1, in a concentration-dependent manner as evidenced by western blot analysis (Figure 6D). In addition, the results also showed that BZBS could inhibit pyroptosis (Figure 6E). Moreover, the inhibitory effect of BZBS on the release of IL-1β and IL-18 was further confirmed by real time PCR (Figures 6F,G).

## 4 Discussion

SAMP8 is a common model for studying accelerated aging (Morley et al., 2012; Cheng et al., 2014). In line with previous studies, we found that the SAMP8 mice had significantly fewer neurons, as shown by Nissl staining (Figures 1A,B) (Yang X. et al., 2020; Yang H. H. et al., 2020; Zhang N. et al., 2022; Zhang Y. et al., 2022). This result, together with the two-photon analysis, indicated that the SAMP8 mice show significant neuroinflammation and BBB disruption (Figure 1C). Moreover, we observed that the intestinal barrier function of the SAMP8 mice was impaired, as evidenced by the damage of the villous structure (Figure 2A). Our previous research has identified a wealth of bioactive compounds in BZBS that directly influence intestinal barrier function and the gut-brain axis (Huang et al., 2021). Chlorogenic acid, a prominent constituent, has been shown to enhance intestinal barrier integrity by alleviating endoplasmic reticulum stress and inhibiting ROCK/MLCK signaling pathways (Song et al., 2022). Additionally, flavonoids found in BZBS have been demonstrated to modulate the gut-brain axis, potentially improving age-related cognitive decline (Chu et al., 2023). These findings highlight the potential of BZBS to exert beneficial effects on intestinal health and cognitive function through its unique blend of bioactive compounds. In this experiment, we confirmed that BZBS treatment improved the integrity of the gut epithelial barrier by increasing the expression of tight junction proteins, and reduced neuroinflammation and enhanced cognitive function (Figures 2–4). Specifically, BZBS reshaped the gut microbiota community in SAMP8 mice, which in turn strengthened the intestinal integrity and suppressed inflammation (Figure 5). Mechanistically, we detected that BZBS treatment markedly prevented the cell pyroptosis by inhibiting the formation of inflammasome in the intestinal tissues of SAMP8 mice (Figure 6).

SAMP8 mice are a common model of accelerated aging that have cognitive and intestinal problems as they age (Yamamoto et al., 2015; Ben Othman et al., 2020; Chen et al., 2021). The gut-brain axis, which is the bidirectional communication between the gut and the brain (Xie et al., 2020; Chen et al., 2021; Zhang N. et al., 2022; Zhang Y. et al., 2022), may be involved in these problems (Xie et al., 2020; Yang X. et al., 2020; Yang H. H. et al., 2020; Yang D. et al., 2023; Yang X. Q. et al., 2023). Many studies have shown that gut dysbiosis, which is the imbalance of the gut microbiota, is associated with not only gastrointestinal diseases, but also the physiology and inflammation of the central nervous system (Ma et al., 2019; Rutsch et al., 2020; Morais et al., 2021). Some studies suggest that the gut dysbiosis may precede and affect the brain health in SAMP8 mice (Pellegrini et al., 2020; D'Antongiovanni et al., 2021). Similar gut-brain connections have been found in other models of Alzheimer’s disease (AD), such as APP/PS1 and 5xFAD mice (Stoye et al., 2020; Guilherme et al., 2021), as well as in normal aging mice (Li T. et al., 2023). Therefore, the gut-brain axis may be a key factor for both cognitive function and longevity (Dumic et al., 2019; Li et al., 2021; Hodge et al., 2022), and SAMP8 mice are a useful model to study how the gut-brain axis works and how drugs that target this axis can improve health and lifespan. We have previously shown that BZBS could effectively improve the cognitive aging caused by D-galactose exposed mice (Ji et al., 2022). Since the gut-brain axis plays an important role in central aging, we wanted to see if BZBS improves central aging through this axis.

Consistent with previous results, we found that SAMP8 mice not only showed increased BBB disruption with age, but also severe intestinal damage (Figures 1, 2). Here, we demonstrated that the SAMP8 mice, as an accelerated aging model, exhibit increased secretion of SASP accompanied by gut dysbiosis (Figure 3), which is consistent with previous studies that aging can lead to gut dysbiosis (Ling et al., 2022). Considering that gut dysbiosis causes central nervous inflammation through the gut-brain axis, we prioritized the detection of gut microbiota changes in the SAMP8 mice, finding a decrease in the ratio of Firmicutes to Bacteroides (F/B) with aging (Vaiserman et al., 2017). Compelling evidence shows that the ratio imbalance of F/B may activate inflammasome in the intestine by increasing the level of LPS, a bacterial toxin (Yu et al., 2017; Paik et al., 2021; Zhang N. et al., 2022; Zhang Y. et al., 2022). Furthermore, the activated inflammasome induces pyroptosis, a type of cell death that releases inflammatory cytokines, and causes intestinal injuries (Kovacs and Miao, 2017). The intestinal barrier damage would allow excessive LPS to enter the bloodstream, fallen into a vicious circle to accelerate systemic aging (Ising et al., 2019; Yang X. et al., 2020; Yang H. H. et al., 2020; de Carvalho Ribeiro and Szabo, 2022; Toldo et al., 2022). BZBS could reshape the decrease in the ratio of F/B and enhance intestinal integrity (Figure 5). Moreover, the KEGG pathways of gut microbiota revealed that LPS biosynthesis proteins was enhanced significantly in SAMP8 mice, and BZBS treatment restored the increased the LPS biosynthesis pathway (Figures 6A,B). LPS translocation owning to intestinal barrier damage was reduced by BZBS by detecting the level of LPS in the liver among groups (Figure 6C).

NLRP3 inflammasome activates pyroptosis, a type of cell death that releases inflammatory cytokines (Elliott and Sutterwala, 2015; Paik et al., 2021). When NLRP3 inflammasome is overactivated, it can cause chronic inflammation in the intestine and disrupt the balance of intestinal cell proliferation and differentiation (Lamkanfi et al., 2007). To further explore the underlying mechanism of BZBS in prevention of intestinal barrier damages, the biomarkers of inflammasome and pyroptosis were measured in each group. We found that BZBS modulated the gut microbiota and its metabolites, and inhibited NLRP3 inflammasome-mediated pyroptosis in the intestine of SAMP8 mice. We measured the expression of NLRP3 and cleaved caspase-1, two markers of inflammasome activation, and the levels of IL-1β and IL-18, two cytokines released by pyroptosis, in each group. We showed that BZBS reduced the expression of NLRP3 and cleaved caspase-1, and the levels of IL-1β and IL-18, in a dose-dependent manner (Figure 6). We also assessed the expression of Ki67 and Lgr5, two indicators of intestinal stem cell activity, and found that BZBS enhanced the processes of intestinal cell proliferation and differentiation (Figure 3). Therefore, our study suggested that BZBS protected the intestine and cognition of SAMP8 mice by suppressing NLRP3 inflammasome-mediated pyroptosis via altering the gut microbiota and its metabolites.

This study explored the therapeutic potential of the compound TCM formula BZBS for age-related cognitive decline. By investigating its effects on the gut-brain axis, we demonstrated that BZBS significantly improved cognitive function and reduced central inflammation associated with accelerated aging. These findings suggest promising therapeutic potential for BZBS in managing age-related cognitive impairment and neuroinflammation. While this study provides valuable insights, it is important to acknowledge its limitations. Our focus on the overall effectiveness based on phenotypes limited the investigation of specific active components and their mechanisms of action. Additionally, a deeper exploration of the advantages of BZBS’s multi-pathway and multi-target approach compared to single-target drugs is crucial to understand its full therapeutic potential. Further research is needed to elucidate the specific active components and their mechanisms of action within BZBS, conduct robust clinical trials to assess the safety and efficacy of BZBS in human populations, and compare the effectiveness of BZBS’s multi-pathway and multi-target approach against single-target drugs for age-related cognitive decline.

## 5 Conclusion

Here, we present a study on the therapeutic effects of BZBS, a traditional Chinese medicine, in intestinal damage caused by accelerated aging. We found BZBS protected the deterioration of the intestinal barrier function by modulating the gut microbiota and its metabolites, and by inhibiting the NLRP3 inflammasome-mediated pyroptosis. These results suggest the therapeutic effect of BZBS on aging-related central diseases, at least partially, targets the gut-brain axis. Meanwhile, our results also confirm the advantages and applications of SAMP8 mice for studying aging-related central aging-related diseases and developing drugs targeting the microbiota-gut-brain axis.

## Data availability statement

The raw sequence data reported in this study have been deposited in the Genome Sequence Archive (Genomics, Proteomics & Bioinformatics 2021) in National Genomics Data Center (Nucleic Acids Res 2022), China National Center for Bioinformation / Beijing Institute of Genomics, Chinese Academy of Sciences (GSA: CRA014256) [https://ngdc.cncb.ac.cn/gsa].

## Ethics statement

The animal study was approved by the ethics commission of the Hebei Yiling Chinese Medicine Research Institute. The study was conducted in accordance with the local legislation and institutional requirements.

## Author contributions

SZ: Data curation, Methodology, Writing – original draft. ML: Methodology, Software, Validation, Writing – original draft. LC: Data curation, Investigation, Writing – original draft. XM: Formal analysis, Project administration, Writing – original draft. YJ: Formal analysis, Project administration, Writing – original draft. XS: Formal analysis, Project administration, Writing – original draft. KN: Formal analysis, Project administration, Writing – original draft. XL: Data curation, Methodology, Writing – original draft. RZ: Formal analysis, Project administration, Writing – original draft. YS: Data curation, Methodology, Writing – original draft. KM: Funding acquisition, Investigation, Supervision, Validation, Writing – original draft. HL: Data curation, Methodology, Writing – original draft. CW: Supervision, Writing – original draft. YH: Conceptualization, Methodology, Writing – review and editing. YW: Conceptualization, Data curation, Funding acquisition, Investigation, Writing – review and editing.
